# High-Resolution Hydrogen–Deuterium Protection Factors from Sparse Mass Spectrometry Data Validated by Nuclear Magnetic Resonance Measurements

**DOI:** 10.1021/jasms.2c00005

**Published:** 2022-04-06

**Authors:** Michele Stofella, Simon P. Skinner, Frank Sobott, Jeanine Houwing-Duistermaat, Emanuele Paci

**Affiliations:** † School of Molecular and Cellular Biology, University of Leeds, LS2 9JT Leeds, United Kingdom; ‡ Dipartimento di Fisica e Astronomia, Università di Bologna, 40127 Bologna, Italy; § Dipartimento di Scienze Statistiche, Università di Bologna, 40127 Bologna, Italy

## Abstract

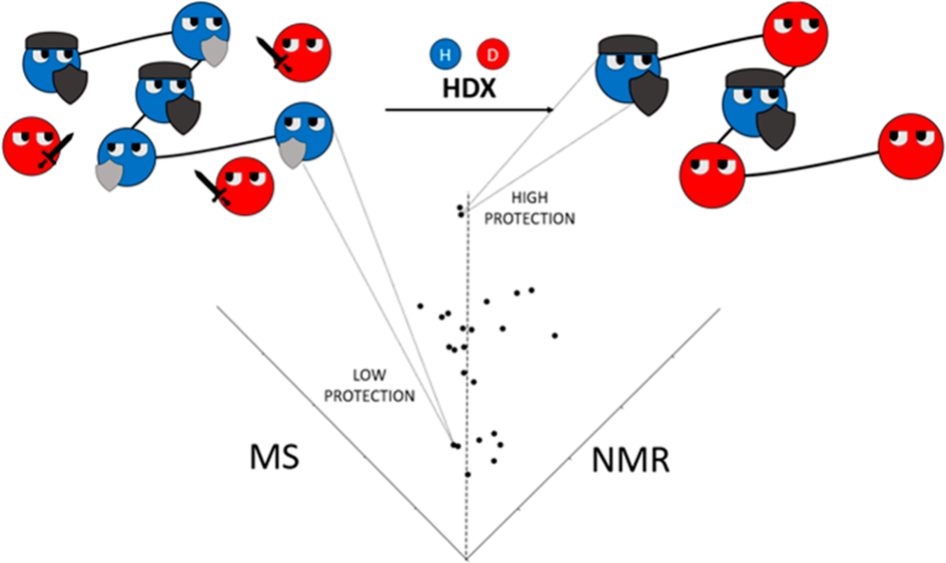

Experimental measurement of time-dependent spontaneous exchange of amide protons with deuterium of the solvent provides information on the structure and dynamical structural variation in proteins. Two experimental techniques are used to probe the exchange: NMR, which relies on different magnetic properties of hydrogen and deuterium, and MS, which exploits the change in mass due to deuteration. NMR provides residue-specific information, that is, the rate of exchange or, analogously, the protection factor (i.e., the unitless ratio between the rate of exchange for a completely unstructured state and the observed rate). MS provides information that is specific to peptides obtained by proteolytic digestion. The spatial resolution of HDX-MS measurements depends on the proteolytic pattern of the protein, the fragmentation method used, and the overlap between peptides. Different computational approaches have been proposed to extract residue-specific information from peptide-level HDX-MS measurements. Here, we demonstrate the advantages of a method recently proposed that exploits self-consistency and classifies the possible sets of protection factors into a finite number of alternative solutions compatible with experimental data. The degeneracy of the solutions can be reduced (or completely removed) by exploiting the additional information encoded in the shape of the isotopic envelopes. We show how sparse and noisy MS data can provide high-resolution protection factors that correlate with NMR measurements probing the same protein under the same conditions.

## Introduction

Hydrogen–deuterium exchange (HDX) is the spontaneous exchange of covalently bonded hydrogens of a protein with deuterium in solution.^1^ In his pioneering work, Lindestrøm-Lang probed the phenomenon through density gradient tubes.^2^ Since then, nuclear magnetic resonance (NMR) has been the leading technique used to probe HDX until the early 2000s,^3^ when mass spectrometry (MS) began to emerge as an alternative with many advantages (no sample size limitations, no labeling required, low protein concentration, low costs, and highly automated processing), counting an increasing number of applications in fundamental biophysics and applied biotechnology.^4−6^ With both NMR and MS, only the exchange of amide hydrogens can be observed because other hydrogens exchange either too fast (side chain acidic and basic hydrogens and polar groups) or too slowly (carbon-bonded hydrogens as well as side chain aliphatic and aromatic hydrogens) to be detected. Hence, in principle, both techniques probe the properties of single amino acids.^7^

NMR exploits the different magnetic properties of hydrogen and deuterium to determine the rate of exchange of individual residues. Their measurement is limited by the resolution of the amide signals themselves, or of cross peaks in homo- or heteronuclear multidimensional NMR spectra.^3^ MS measures directly the mass variation as a function of exchange time of peptides obtained by proteolytic digestion.^4^ The spatial resolution of HDX-MS measurements depends on the digestion pattern of the protein, the overlap between peptides, and the MS/MS fragmentation methods used.^8^ Most current approaches use collision-induced dissociation (CID) for MS/MS fragmentation of peptides, but because of H/D scrambling during collisional activation, no information is gained on the exact location of deuterium labels within the peptides. Instead, such MS/MS data merely serve to unambiguously identify peptides by their sequence tags. Different approaches have been proposed to increase spatial resolution, including the use of alternative MS/MS methods, which minimize H/D scrambling during fragmentation.^9−11^

Several computational strategies have been proposed to extract single-residue protection factors from peptide-level HDX-MS data.^12−19^ Here, we demonstrate the advantages of a method recently proposed^20^ that exploits self-consistency (i.e., data consistency among overlapping peptides) and finds alternative sets of protection factors equally consistent with experimental data. These solutions can be classified into a finite number of clusters whose degeneracy can be further reduced by exploiting the additional information contained in the shape of the isotopic envelope. We show how sparse and noisy MS data can provide high-resolution protection factors that correlate with NMR measurements probing the same protein at the same conditions.

The exchange kinetics of an amide proton is highly dependent on the environment, hence, a unique probe of the structure and dynamics of proteins. Since the seminal work from Linderstrøm-Lang,^2^ HDX has been modeled as a two-step process. The deuteration of a residue in a D_2_O solution is possible if a local *opening* of the structure occurs:
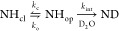
1Here, *k*_o_ and *k*_c_ refer to *opening* and *closing* rates, respectively, which allow the residue to switch from an exchange-incompetent state (i.e., a closed or folded state NH_cl_) to an exchange-competent state (i.e., an open or unfolded state NH_op_). The intrinsic exchange rate *k*_int_ is the exchange rate of the residue in a completely unstructured protein and depends on the pH, temperature of the solution, and side chains of the two adjacent amino acids.^21−25^

Since, for a folded protein *k*_c_ ≫ *k*_o_ (native state approximation), the observed exchange rate can be written as
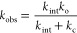
2This expression suggests two limiting cases depending on the relative size of *k*_int_ and *k*_c_. If *k*_int_ ≪ *k*_c_ (EX2 regime), the deuteration of a single residue is
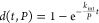
3where the opening equilibrium constant *P* ≡ *k*_c_/*k*_o_, known as the protection factor, is linked to the dynamic properties of the residue by definition; moreover, several studies have shown a correlation between the protection factors of a protein and its structure.^26,27^ If instead *k*_c_ ≪ *k*_int_ (EX1 regime), *k*_obs_ = *k*_o_. Under physiological conditions, the EX2 regime dominates the exchange kinetics in natively folded proteins.^28^

In HDX-NMR experiments, the proton signal decays exponentially as deuteration occurs because deuterium is ^1^H NMR silent, and the experimental curves can be fitted with eq 3 to obtain *P*.^29,30^

On the other hand, HDX-MS measures the exchange of proteolytic peptides, with experimental curves resulting in a sum of exponentials. The fractional deuterium uptake at time *t* of a peptide of *N* exchangeable residues (i.e., excluding prolines and the *N* terminus) is
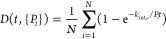
4where *P*_*i*_ and *k*_int,*i*_ are the protection factor and the intrinsic exchange rate of the residue *i*. If exchange rates (or, equivalently, protection factors) are known for each residue, the exchange kinetics of peptides is uniquely defined, but not vice versa.^20^

The possibility of estimating individual protection factors from HDX-MS data depends on four factors:^8^ (i) peptide overlap, (ii) time point resolution, (iii) time window coverage, and (iv) experimental error. (i) The protection factor of an individual amino acid can, in principle, be extracted only if two proteolytic peptides differ by exactly one amino acid. When multiple peptides partly overlapping are available, protection factors are ambiguous, with the ambiguity decreasing with an increase in the number of overlapping peptides.^12,20^ In the case of “exact” measurements (i.e., not affected by experimental error), the problem is combinatorial: for an isolated peptide formed by *N* residues, there are *N*! possible solutions (Supplementary Figure 1A); for two overlapping peptides formed by N1 and N2 residues, respectively, and with *N*_c_ residues in common, there are (N1 – Nc)!(N2 – Nc)!Nc! alternative solutions (Supplementary Figure 1B). Reporting a solution in terms of observed rates (*k*_obs_ = *k*_int_/*P*) or protection factors yields equivalent results with different numerical values arising from the different intrinsic exchange rates between residues (Supplementary Figure 1C). Although the observed rates span several orders of magnitude depending on the experimental conditions (pH, temperature), the protection factor can be restricted to the boundaries 0 < ln(*P*) ≤ 20, facilitating the convergence of fitting algorithms. (ii) The fractional uptake of a peptide (eq 4) is measured for a discrete set of times (*N*_times_); if these are fewer than the exchangeable amino acids in the peptide (*N*_res_), the individual residues’ protection factor is underdetermined: multiple solutions are equally consistent with experimental data. Even for small peptides, though, where in principle the number of time points is sufficient to extract all the exchange rates (*N*_res_ ≪ *N*_times_), the solutions are degenerate because eq 4 does not contain information on the relative contribution of the fitting parameters (protection factors). A necessary condition, albeit not sufficient, is that the number of experimental points should be no less than some multiple *Q* (quality factor) of the number of adaptive parameters in the model:^31^*N*_exp_ ≈ *QN*_res_, where *N*_exp_ = *N*_times_ for an isolated peptide. (iii) To properly sample the multiexponential uptake of a peptide (eq 4), these exchange times should follow a log-uniform distribution between the *beginning* and the *end* of the exchange process, which can be deduced from the exchange of the whole protein (i.e., without digestion). Typical HDX-MS measurements report time-resolved exchange between tens of seconds and hours. The detection of exchange at shorter times (e.g., subsecond) is now possible, with recent developments giving access to millisecond time scales.^32−34^ A simultaneous fitting of the information encoded in multiple overlapping peptides reduces the degeneracy on the rate-to-residue assignment by adding local information. Moreover, it increases the number of experimental points *N*_exp_: for a region formed by *N*_res_ residues and covered by *N*_pep_ peptides, *N*_exp_ = *N*_times_*N*_pep_. Experimentally, the overlap of peptides depends on the choice of the protease, which is limited because of the acidic conditions needed to quench the exchange. (iv) The presence of experimental uncertainties affects the accuracy on the final predictions. The law of large numbers ensures that the average value among independent measurements (replicates) tends to the mean of the measurements, that is, the true value of the estimated quantity in the limit of an infinite number or replicates. The number of replicates provided in HDX-MS experiments (generally three) limits the accuracy of the measured quantity (i.e., the fractional uptake), and consequently of the estimated protection factor.

Two computational approaches aim to extract protection factors at the highest resolution possible from HDX-MS data sets. HDSite^12,35^ uses the isotopic envelopes to derive the extent of deuteration of each residue of the peptide at different exchange times (0 ≤ *d* ≤ 1), and the obtained curve can be further fitted with a single exponential (eq 3) to obtain the protection factor. An initial guess on the deuteration of each residue is refined to reproduce the isotopic pattern. The probability of exchange for a residue follows a binomial distribution where the “success probability” is given by the deuteration of the residue and is therefore a function of time. Hence, the isotopic pattern can be calculated as the product of binomial probability distributions (one per amino acid) further convoluted with the natural abundance of elements. In practice, HDSite derives single residue protection factors only when the uptake of a residue can be calculated as the difference in uptake of two peptides, otherwise an averaged value is returned. Therefore, the method strongly depends on the data set, and the prediction is limited by the number of peptides available and their overlap. Analogously to HDSite, other methods aim to extract single residue information from HDX-MS data by fitting the isotopic envelopes of peptides.^15−17^ A method more recently proposed (ExPfact)^20^ simultaneously fits the uptake curves of contiguous overlapping peptides with multiexponential curves (eq 4), determining all alternative patterns of protection factors compatible with experimental data. This method can be applied to any data set, and the ambiguity on the predicted protection factors provides a measurement of the degree of underdetermination of single residue properties. A similar approach has been implemented by pyHDX,^13^ HDXModeller,^14^ HR-HDXMS,^18^ and HDX Workbench.^19^

In this article, we analyze a data set previously published,^36^ containing sparse HDX data from MS and NMR measurements under the same experimental conditions for the small monomeric mouse prion protein (103 amino acids). Using ExPfact,^20^ we show that a discrete number of sets of protection factors can be extracted from sparse HDX-MS data, that the ambiguity on the estimate can be reduced when a proper temporal sampling is coupled with minimal overlap, and completely removed by exploiting the additional information contained in the isotopic envelopes *a posteriori*. The extracted protection factors correlate with NMR measurements, with discrepancies providing insights on the compatibility between the two techniques as well as strengths and limitations of the statistical approach implemented.

## Methods and Materials

### Data Set

**Figure 1:**
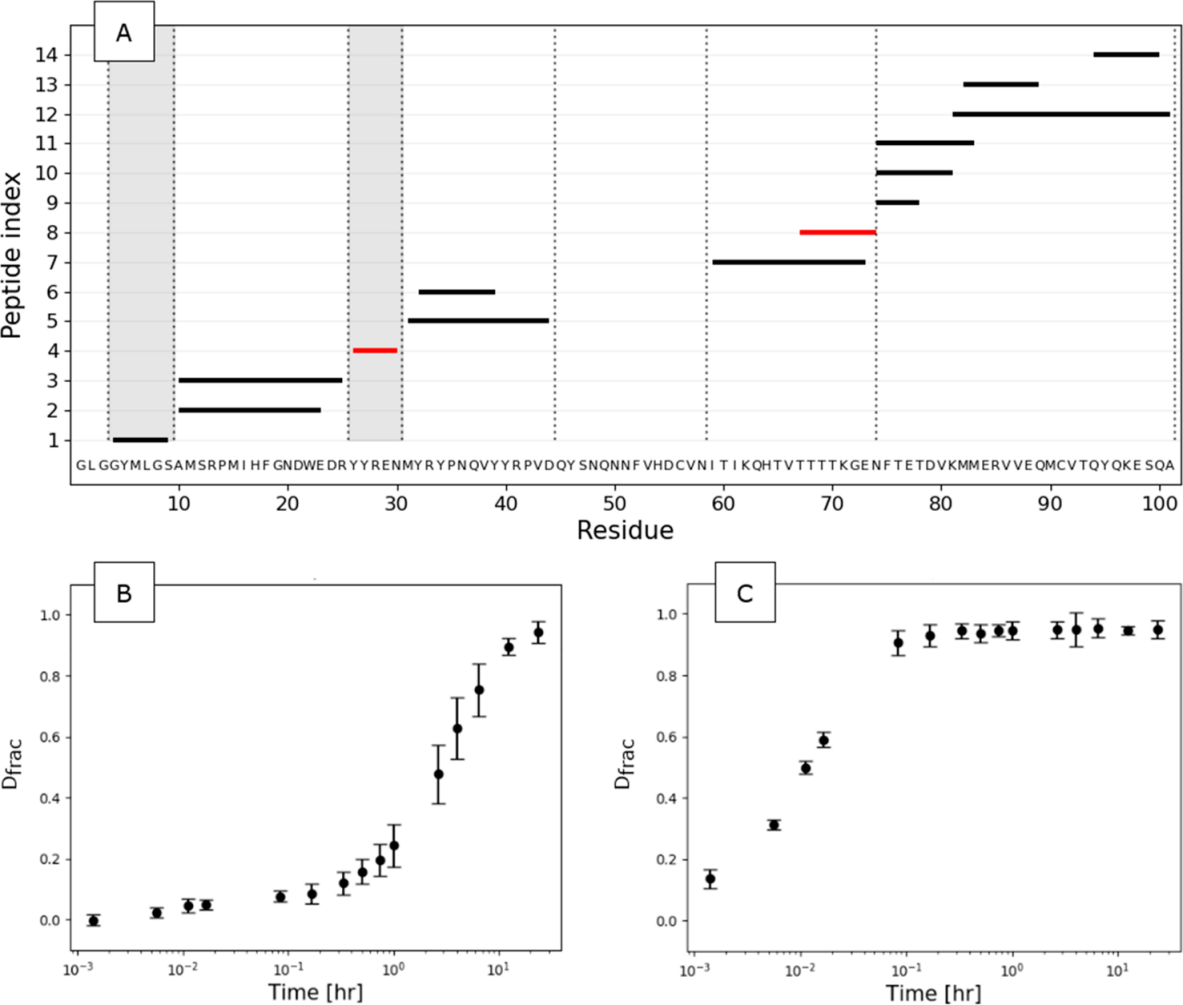
HDX-MS data set previously published in ref (36). (A) The coverage map localizes the 14 peptides identified after pepsin digestion. The six regions covered by isolated (gray) or contiguous overlapping peptides are separated by vertical dotted lines. (B, C) The fractional uptake of peptides 4 (B) and 8 (C)—highlighted in red in the peptide map—is shown at 15 time points.

The measurements analyzed here were previously published^36^ and probed the mouse prion protein (103 residues) at pH 4 and a temperature of 25 °C at different urea concentrations. To ensure the validity of the EX2 approximation (and thus of eq 3 and eq 4), we focused on the exchange of the protein in its native state (i.e., in the absence of urea). In the HDX-MS experiment, the exchange was quenched at pH 2.4 and a temperature of 0 °C, and the protein was digested by pepsin, providing a data set (Figure 1) that includes 14 peptides covering most of the sequence (75/103 residues were covered) but with marginal overlap. Six regions covered by contiguous overlapping peptides were identified. The exchange was monitored at 15 exchange times ranging from 5 s to 24 h, and the experiment was conducted in triplicate. Data are available in the Supporting Information.

The fractional deuterium uptake of a peptide *D* was calculated as the intensity-weighted average (centroid) of the isotopic envelope at a specific time *D*_*t*_ and was normalized using the centroid of the experimentally fully deuterated sample *D*_FD_ (which was lower than the theoretical, fully deuterated centroid because of back exchange) and the centroid of the fully protonated sample *D*_0%_:
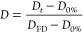
5The fractional uptake was then averaged over the three replicates.

HDX was also measured by NMR under the same experimental conditions, and exchange rates were derived for 34 amino acids. A subset of 27 residues was covered by both data sets. Because NMR experiments were performed only once, we assumed that the protection factors provided by NMR represent their true values.

### Prediction of Protection Factors from HDX-MS Data

ExPfact is a computational method aiming to extract protection factors at the resolution of the single amide.^20^ Considering regions covered by contiguous overlapping peptides one by one, the method finds multiple solutions of a system of equations (the size of which depends on the number of overlapping peptides in each region, and each equation has the functional form in eq 4), and then clusters these, reducing the degeneracy and providing a discrete number of alternative averaged solutions.

**Figure 2:**
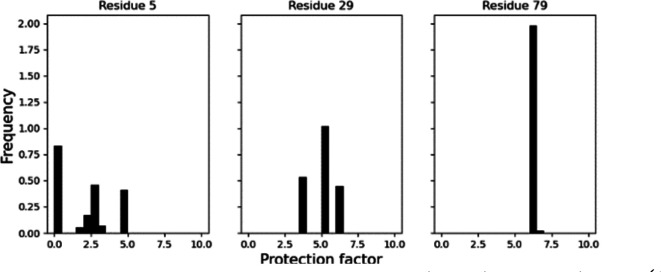
Histograms of protection factors predicted for residues 5 (left), 29 (center), and 79 (right) from 5000 minimizations. In most cases, histograms are multimodal distributions: Three modes can be identified for residue 5; 4 modes for residue 29.

To find one possible solution, we performed a best fit on the experimental data. The experimental fractional uptake *D*_*j*_^exp^ was simultaneously fitted for every peptide *j* at every time point *t*_*k*_ with eq 4 (*D*_j_^pred^), and the set of protection factors {*P*_*i*_} was adjusted to minimize the cost function
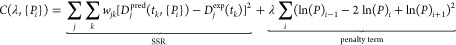
6The cost function in eq 6 consists of a regular term, the sum of squared residuals (SSR), which depends on the experimental data, and a penalty term, which was introduced to avoid overfitting and, given the correlation between exchange rates and structure of the protein,^26,27^ to disfavor large variations in the protection factors of adjacent residues. Gaps between peptides and prolines do not influence the penalty term, which is set to 0 unless ln *P*_*i*–1_, ln *P*_*i*_, and ln *P*_*i*+1_ are simultaneously greater than 0 (ln(*P*) is set to −1 for prolines and for any residue not covered by peptides). The penalty constant was set to λ = 10^–8^ after cross validation (Supplementary Figure 2). Following the recommendations for the propagation of error in HDX-MS data,^37^ a pooled standard deviation can be associated with each measure; therefore, the weights *w*_*jk*_ are all equal. When reliable error estimates are available—which is unlikely the case when the number of replicates is limited to three—then it is more accurate to consider the weights as the inverse of the standard deviation. The cost function in eq 6 represents a rough fitting landscape, and depending on the initial guess for the set {*P*_*i*_}, the minimization algorithm converges to different local minima. When not specified, the initial guess is chosen through a random search: 10 000 sets of protection factors are randomly initialized with the constraint 0 < ln(*P*) ≤ 20, and the set with the best agreement with experimental data (i.e., with the lowest cost function) is selected as the initial guess for a least-squares minimization. To explore alternative local minima in the fitting landscape, and thus to calculate several possible solutions, this minimization procedure is repeated 5000 times. To reduce the degeneracy of the sets of protection factors, we applied a clustering algorithm based on Gaussian mixture models (GMM), implemented in the R package mclust.^38^ The histograms of the predicted protection factors, which are often multimodal (Figure 2), are combined into an *M*-dimensional probability distribution (*M* being the length of the region covered by overlapping peptides), which is fitted with a mixture of Gaussians with variable means and covariances (Supplementary Figure 3). The clustering algorithm returns a finite number of clusters of sets of {*P*_*i*_}, each one in agreement with HDX-MS experimental data. The final number of identified clusters is determined by BIC (Bayesian information criterion). The minimization procedure is repeated until the addition of new solutions does not alter the outcomes of the clustering algorithm.

### Performances

One minimization procedure requires on average 12.7 s on the data set analyzed here (processor: Intel Xeon W-1290P 3.7 GHz) using the default tolerance parameter (--tol), which controls the convergence of the algorithm. To speed up the process, the code was parallelized to run on multiple cores (parameter --ncores). Splitting the calculations over four cores is sufficient to complete 5000 minimizations in less than 5 h. We recommend running ExPfact overnight and setting up the number of minimizations and the tolerance parameter according to the computational power available.

### Prediction of Isotopic Envelopes

For a peptide, the fractional deuterium uptake at time *t* (Figure 1B,C) is the mean of the centroids of the isotopic envelopes of different replicates. However, the same centroid value corresponds to different isotopic envelopes depending on the deuterium uptake of individual amino acids. Isotopic envelopes estimated from a predicted set of protection factors provide additional information to select the correct solution among all those that fit the time evolution of the centroid of each isotopic envelope.

To simulate the time evolution of the isotopic envelope of a peptide formed by *n* exchangeable residues, we need to calculate the probability that *k* residues have exchanged at time *t*:
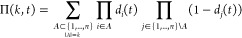
7Equation 7 can be built following these considerations: (i) The probability of a residue to exchange is a function of time and is given by eq 3; (ii) the probability of *k* residues to have exchanged is the product of their individual probabilities (assuming they are independent events); (iii) the probability that only *k* residues of an *n*-residue peptide have exchanged is given by the probability that *k* residues have exchanged times the probability that *n* – *k* residues have not exchanged; (iv) the calculations in points (i)–(iii) must be summed over all possible combinations of *k* residues in the *n*-residue peptide.

The isotopic envelope of a peptide can be calculated by applying the evolution in eq 7 to the fully protonated envelope of the peptide (calculated using the python library pyOpenMS^39^). Given the intensity of the fully protonated envelope π_*i*_ for a species with isotope number *i*, the simulated intensity of the isotopic envelope at time *t* is given by π_*i*_Π(*k*=0,*t*) + π_*i*–1_Π(*k* = 1,*t*) + ... + π_*i*–*N*_Π(*k*=*N*,*t*) = ∑_*j* = 0_^*N*^π_*i*–*j*_Π(*j*,*t*), where the species *i* – *N* corresponds to the monoisotopic mass of the peptide.

**Figure 3:**
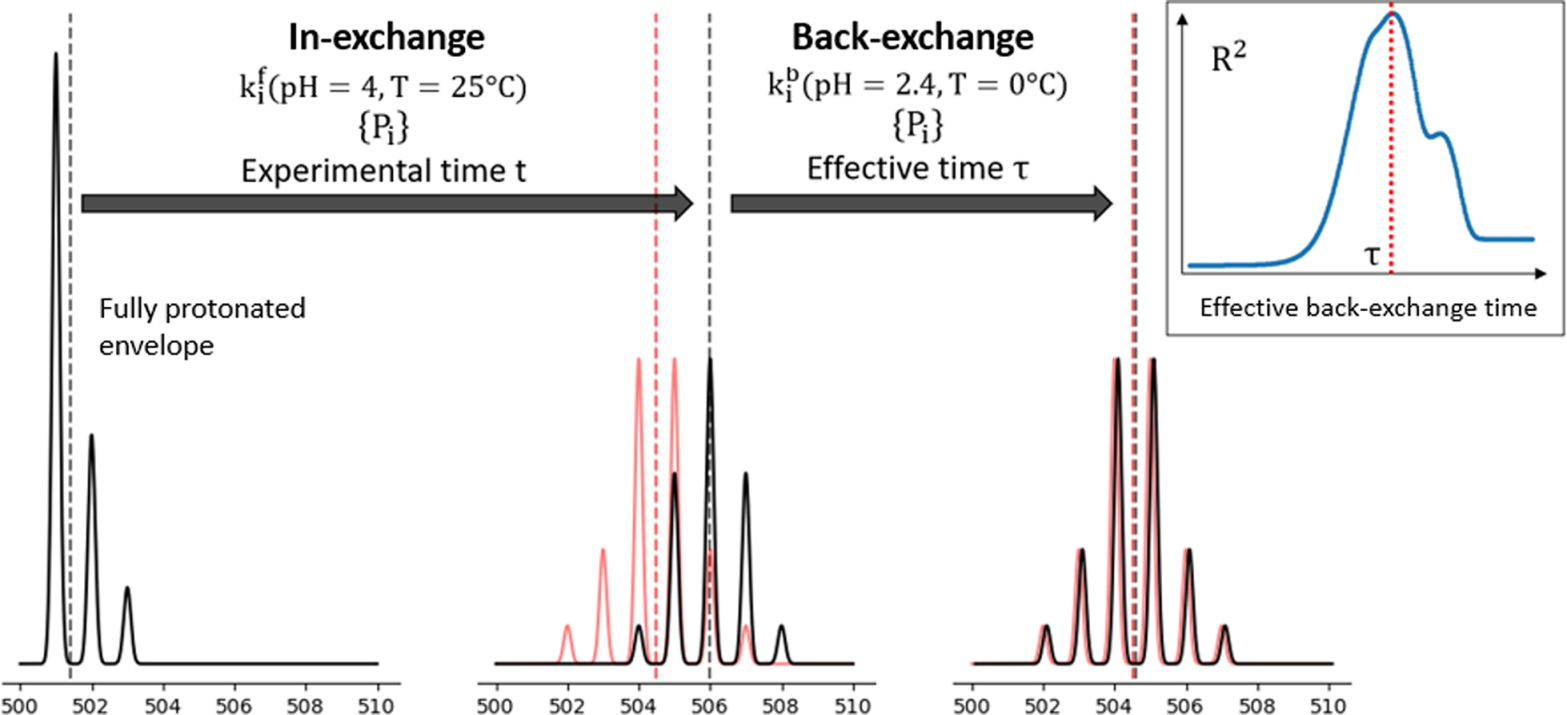
Schematic representation of the calculations for the reproduction of the experimental isotopic envelope. The fully protonated envelope can be calculated from the knowledge of the peptide sequence. The isotopic envelope at time *t* is evaluated by applying the evolution in eq 7 to the fully protonated envelope, using a specific pattern of protection factors {*P*_*i*_} and the intrinsic “forward” exchange rates *k*_*i*_^f^ calculated at pH 4 and a temperature of 25 °C for a protonated protein in a deuterated buffer. The in-exchange predicts an envelope (black) that lies at higher *m*/*z* values with respect to the experimental spectrum (red); vertical dashed lines indicate the centroid of the envelopes. To correct for back exchange, the evolution in eq 7 is applied toward protonation, using the same {*P*_*i*_} and the intrinsic “back”-exchange rates *k*_*i*_^b^ calculated under quenching conditions (pH 2.4 and temperature 0 °C) for a deuterated protein in water. The back-exchange evolution is applied for an effective back-exchange time τ, which maximizes the agreement between the predicted and the experimental envelope (insert).

To calculate the shape of the isotopic envelope at time *t* from a set of {*P*_*i*_}, the evolution in eq 7 was applied until deuteration time *t*, that is, toward higher *m*/*z* values, using the intrinsic exchange rates calculated at a temperature of 25 °C and pH 4, the conditions at which the experiment was performed, for a protonated protein in a deuterated buffer. However, the predicted envelope always appeared at higher *m*/*z* values with respect to the experimental ones because the deuteration in eq 7 does not account for back exchange. Back exchange occurs at the protein level in the labeling buffer, which is never 100% deuterated (generally 90–95% D_2_O), and in the quench buffer before injection into the pepsin column, after which back exchange also occurs at the peptide level. To reproduce the shape of the isotopic envelope, we applied the evolution in eq 7 toward protonation, that is, toward lower *m*/*z* values, using the same set of {*P*_*i*_} and the intrinsic exchange rates calculated at a temperature of 0 °C and pH 2.4 for a deuterated protein in a protonated buffer. This back-exchange correction was applied for the “effective back-exchange time” τ that minimizes the difference between the predicted and the experimental shape. The underlying assumption is that back exchange can be modeled analogously to “in exchange” (i.e., using the multiexponential in eq 4). We used the predicted envelopes to discriminate whether some pattern of protection factors was able to better reproduce the shape of the isotopic envelope; the agreement was evaluated with R^2^. The procedure for the prediction of isotopic envelopes is summarized in Figure 3.

## Results

**Figure 4:**
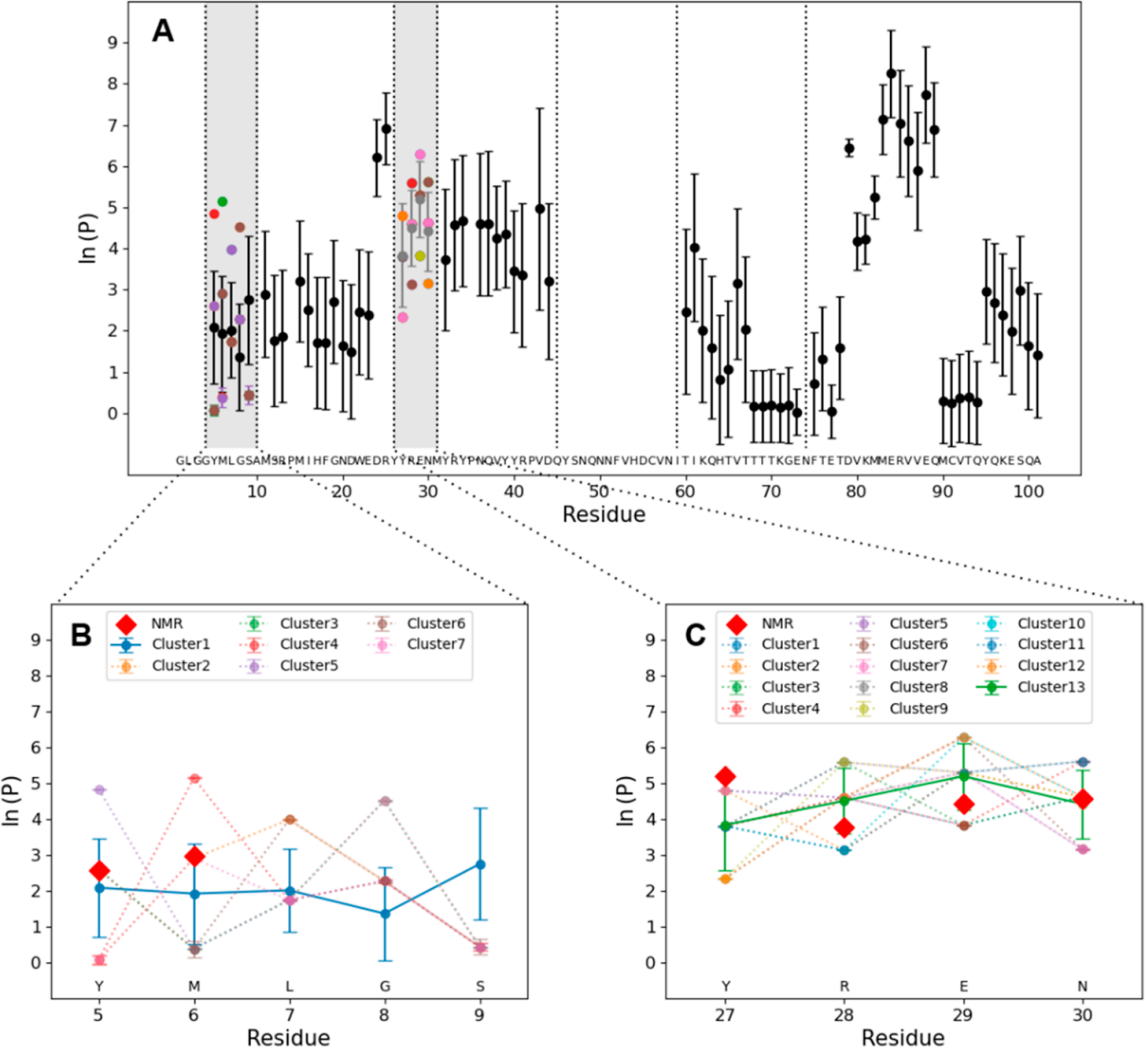
(A) Protection factors corresponding to cluster means of 5000 least-squares solutions obtained by minimizing the cost function in eq 6. Vertical dashed lines show regions covered by isolated (gray) or overlapping peptides (compare to Figure 1). Dots and error bars represent the mean and standard deviation of the estimated clusters. In regions where multiple clusters are identified, different clusters are shown with different colors. (B, C) Comparison of the estimated clusters with protection factors from NMR (red diamonds) in the regions where multiple clusters are identified; clusters compatible with NMR measurements, namely, clusters 1 and 13 in the regions covered by residues 5–9 and 27–30, respectively, are highlighted.

The protection factors derived from HDX-MS measurements probing the mouse prion protein in its native state are shown in Figure 4A with their associated error. The value(s) and the error(s) associated with protection factors derived from MS measurements are the mean(s) and standard deviation(s) of the Gaussian cluster(s). For most of the sequence, a single cluster is found (i.e., all possible solutions correspond to a single cluster, see Methods and Materials), whereas multiple clusters are found in the two regions (residues 5–9 and 27–30) in which proteolytic peptides do not overlap (Figure 4B,C). In both regions, the NMR protection factors fall within 1σ (1 standard deviation) of the MS estimation. The predicted profiles of protection factors reflect the known structural properties of the protein. Indeed, higher protection against exchange is observed at helices α1 (residues 21–30) and α3 (residues 77–101), with completely unprotected residues surrounding Cys91, which forms a disulfide bond with Cys56. Lower protection is also observed in the loop between α2 and α3 (residues 72–76).

**Figure 5:**
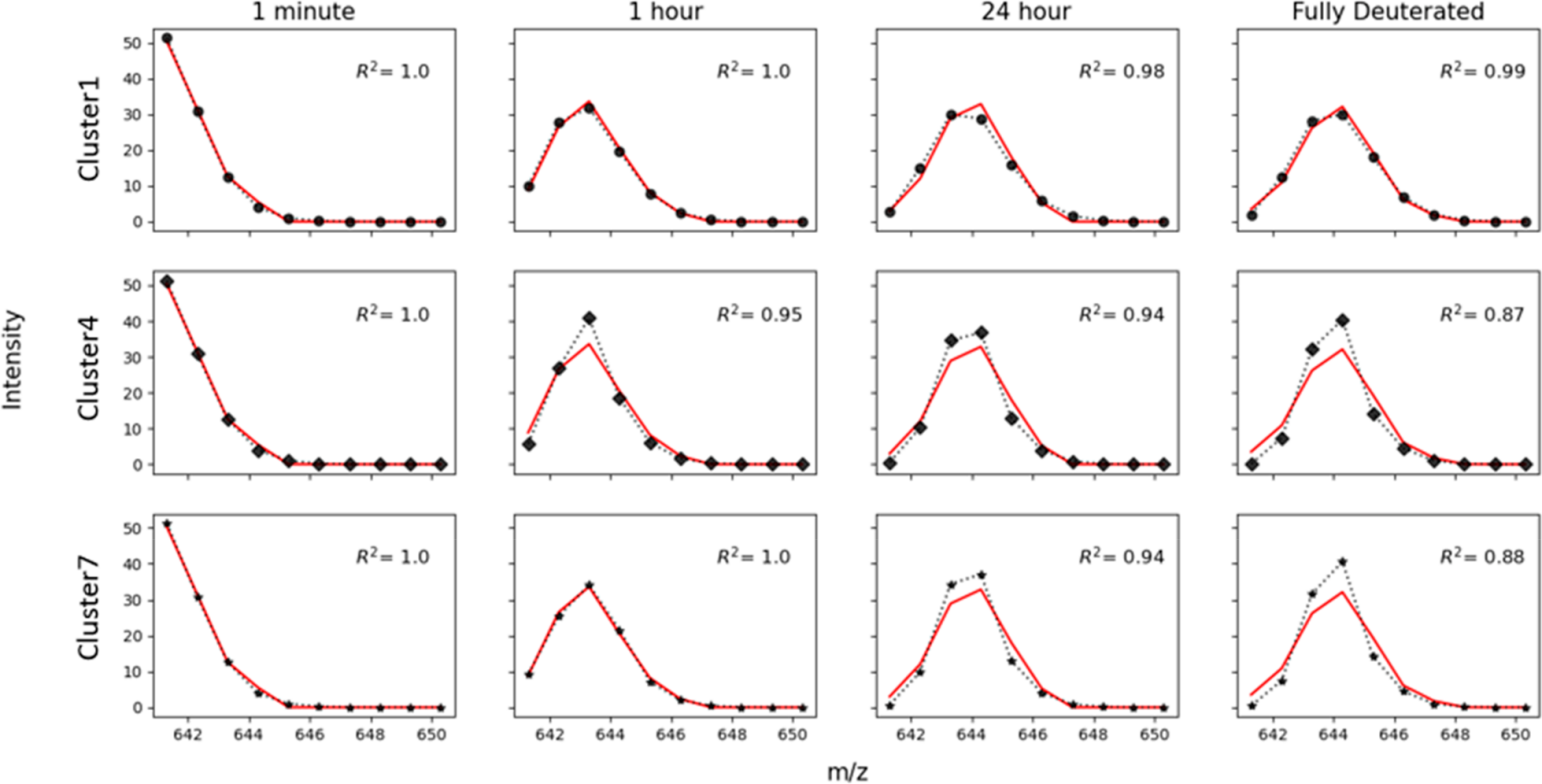
Prediction of isotopic envelopes. Starting from the fully protonated envelope of peptide 1 (sequence YMLGSA), the evolution in eq 5 is applied toward deuteration at times 1 min (column 1), 1 h (column 2), 24 h (column 3), and infinite time (column 4) using intrinsic exchange rates calculated at pH 4 and a temperature of 25 °C and a set of protection factors belonging to cluster C1 (row 1), C4 (row 2), and C7 (row 3). A back-exchange correction is performed by applying eq 5 toward protonation, using intrinsic exchange rates calculated at pH 2.4 and a temperature of 25 °C and the same set of protection factors. The isotopic envelope predicted using protection factors from Cluster1 (black dots), Cluster4 (black diamonds), and Cluster7 (black stars) is compared to the experimental envelopes (red lines). The agreement is evaluated using R^2^.

The shape of the experimental isotopic envelope can be exploited to define the quality of each cluster of solutions. We show the results for peptide 1 (residues 4–9, Figure 1), where the clustering algorithm identified seven clusters (Figure 4). We randomly select a set of {*P*_*i*_} from each cluster and predict the isotopic envelope as discussed in the Methods and Materials section. The outcomes (Figure 5) show that the solutions belonging to cluster 1, which was the only cluster compatible with NMR measurements, can reproduce the shape of the experimental isotopic envelope better than any other cluster. This proves that the isotopic envelopes encode a greater amount of information relative to centroided data, and that this information can be used *a posteriori* to reduce the ambiguity on the estimated value of protection factors.

**Figure 6:**
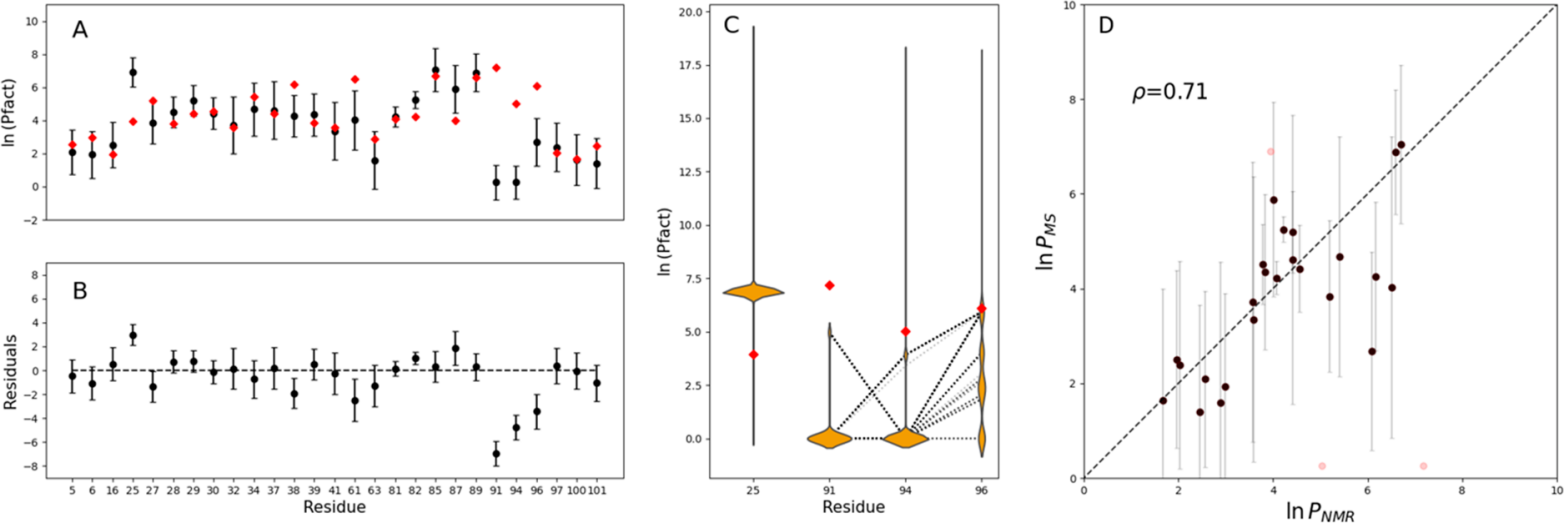
Comparison of protection factors from HDX-MS and HDX-NMR experiments. (A) Clusters of protection factors extracted from HDX-MS data (black dots with error bars) are compared to NMR measurements (red diamonds) for every amino acid covered by both data sets. (B) Residuals of protection factors from HDX-MS and HDX-NMR experiments. (C) Marginal probability distribution of protection factors derived from 5000 minimization procedures for residues 25, 91, 94, and 96. (D) Correlation between protection factors extracted by NMR and MS; Pearson’s correlation coefficient ρ = 0.71 excluding outliers (red).

Protection factors for 27 residues covered by the MS data set are also available from NMR measurements. We were able to extract single residue protection factors from MS centroided data for all but two regions (Figure 4). Moreover, we were able to assess the quality of different solutions in one of these regions, therefore deriving one “top-scoring” pattern of protection factors (Figure 5). The region covered by peptide 4 (Figure 1) remains underdetermined (Figure 4C) because the experimental isotopic envelopes for this peptide were not available. In this region, we selected cluster 13 as the final pattern of protection factors because it showed compatibility with NMR measurements. The comparison between protection factors extracted by MS and NMR (Figure 6A,B) showed a high degree of compatibility between the protection factors extracted by the two techniques, with 23/27 values compatible with at most 2σ, and a correlation coefficient ρ = 0.71 (Figure 6D) when the outlier residues 25, 91, and 94 (which are not compatible within 3σ) are not considered.

## Discussion

Despite the only partial coverage provided by the MS data set (Figure 1), we showed how alternative patterns of protection factors with similar agreement to experimental data can be accurately derived at the resolution of a single amino acid (Figure 4A). Moreover, the solutions can be clustered, providing a discrete number of alternative solutions for {*P*_*i*_}. In most regions, one unique cluster was identified. Two regions still present ambiguity in the final estimate of the protection factors, but at least one of the clusters identified in these regions is compatible with protection factors derived from NMR measurements (Figure 4B,C). Nonetheless, the ambiguity could be completely removed for one of these two regions by exploiting the supplementary information contained in the shape of the experimental isotopic envelope (Figure 5). Therefore, the method used here estimates protection factors from MS data alone (with the exception of the region covered by residues 27–30, where the experimental isotopic envelope was not available).

A comparison of the protection factors estimated from MS with measures from NMR showed a high degree of compatibility (Figure 6), validating the method. The four discrepancies shown by residues 25, 91, 94, and 96 provide insight into the limitations of the data sets and the computational approach. The protection factor of residue 25 is compatible within 3σ with the NMR measurement. Interestingly, the marginal probability distribution of the protection factors estimated for residues 91 and 94 is bimodal, with one of the modes similar to the NMR measurements (Figure 6C). The GMM clustering algorithm selects the final number of components based on the minimum BIC = *k* ln(*n*) – 2 ln(*L̂*), where *n* is the number of data points, *L̂* the maximized value of the likelihood function of the model, and *k* the number of parameters estimated by the model. Therefore, the BIC tends to favor models with fewer parameters. Considering the low-intensity peaks as outliers of the main distribution leads to a lower BIC than considering them as separate modes of a multimodal distribution. This artifact is even more evident when we look at protection factors of residue 94, which has a multimodal probability distribution with four modes; moreover, one of the modes is compatible with the NMR measurement. Even in this case, the BIC is lower when the projected multimodal distribution is merged into one component.

**Figure 7:**
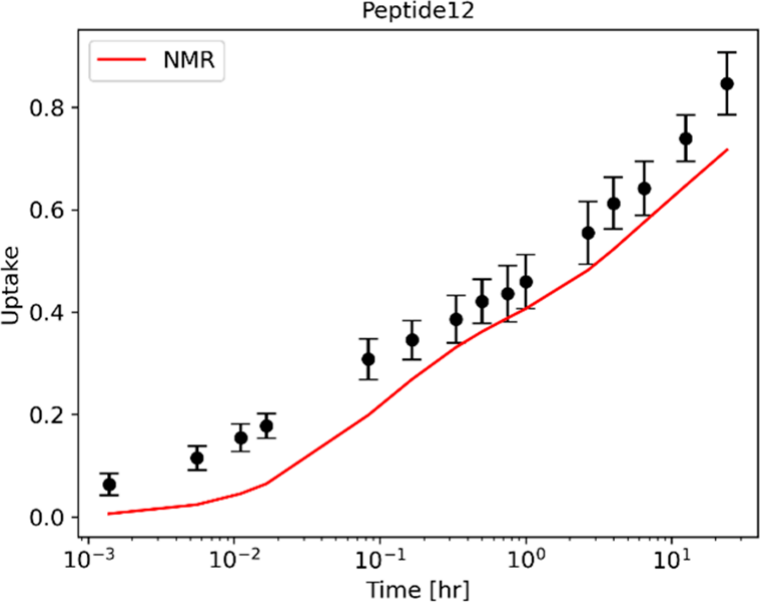
Deuterium uptake prediction for peptide 12 using an optimized set of protection factors with constrained NMR values. In the region covering residues 76–101, the protection factor of 11 residues was measured by NMR. These values are fixed, whereas the remaining protection factors are optimized to minimize the cost function in eq 6. The resulting prediction (red line) is not compatible with MS data.

A univariate clustering approach (i.e., a clustering algorithm considering one residue at a time instead of regions covered by contiguous overlapping peptides, Figure S3) would find the low-intensity peaks approaching NMR measurements shown in Figure 6C for residues 91, 94, and 96. However, a multivariate approach is statistically and physically more rigorous because the protection factor of a residue depends on its neighbors. Indeed, there is not a single pattern of {*P*_*i*_} found by the minimization procedure containing simultaneously all those three values (black dotted lines in Figure 6C show the subset of solution with 4 < ln(*P*_91_) < 6 or 2.5 < ln(*P*_94_) < 5). Moreover, a set of {*P*_*i*_} with protection factors of residues 91, 94, and 96 equal to NMR measurements did not fit the uptake curves of the HDX-MS data set. To prove this, we constrained protection factors in the region 75–101 to their NMR value (when available) during the least-squares minimization, whereas the remaining protection factors are adjusted to minimize the cost function in eq 6. For peptide 12, which contains residues 91, 94, and 96, a best fit provides a prediction in deuterium uptake, which is not compatible with MS measurements (Figure 7). The analysis of these discrepancies suggests that an estimation of the same quantity (i.e., the protection factor) from two different techniques is not possible here because the error is either unknown (in the NMR data set) or too large (in the MS data set). The disagreement is however localized in a specific region of the protein and could therefore be caused by artifacts in either the NMR or MS experiment. In the absence of additional measurements, these results cannot be interpreted further.

## Conclusions

In this article, we applied ExPfact^20^ to a previously published data set by probing the HDX of the same protein under the same experimental conditions by both MS and NMR.^36^ The novelties introduced with respect to the previous publication are (i) the validation of the method via a comparison to NMR data, which is often neglected in related papers;^12−19^ (ii) the prediction of the experimental isotopic envelope of peptides (via the back-exchange correction) as another tool to assess the quality of alternative solutions; (iii) several upgrades to the code (introduction of the penalty term, parallelization of the code, additional scripts and tests, and extended documentation).

The approach demonstrated here enables the quantitative analysis of any HDX-MS data set (in the EX2 regime), providing protection factors at the resolution of the single amino acid. We note that the protection factor is a well-defined quantity only when both the native and EX2 approximations are valid. When EX1 kinetics (or mixed EX1/EX2 kinetics) emerge from the isotopic distribution of peptides, single residue information can be extracted via other methods.^17,40^ The information extracted in the two regimes is different. For the exchange in EX2 conditions, a protection factor can be extracted: this is a unitless quantity that can be expressed with Gibb’s free energy of opening: Δ*G*_op_ = *RT* ln *P* (where *R* is the universal gas constant and *T* is the temperature).^1^ In the case of EX1 kinetics, the exchange of a single residue is *d*_EX1_(*t*) = 1 – e^–*k*_o_*t*^; therefore, it is possible (in principle) to extract the opening rate *k*_o_ of a residue, which has the units of [time]^−1^ and can be expressed through the Eyring equation^41^ as proportional to Gibb’s free energy of activation:  (where *k*_B_ is the Boltzmann constant and ℏ is Planck’s constant).^7^ ExPfact aims to extract protection factors from HDX-MS because they encode structural information on the protein, and is consequently limited to the study of data sets with peptides showing EX2 behavior.

HDX-MS is a promising technique for high-throughput and low-cost characterization of proteins’ structural and dynamic properties. The principal drawback of the technique is its spatial resolution, providing data at the peptide level, which so far are mostly interpreted qualitatively. The implementation of alternative MS/MS fragmentation methods not affected by H/D scrambling—such as electron capture/transfer dissociation (ExD) and UV photodissociation (UVPD)^9−11^ —would be a valuable addition to experimentally increase spatial resolution. However, we believe that single residue resolution will be hardly achieved for the whole sequence of the protein. Therefore, computational methods aiming to extract information at higher resolution will remain essential. The efforts made by the HDX-MS community to acquire higher quality data^32−34^ combined with a unified computational approach encompassing the knowledge acquired in the past decade^12−20^ will enable HDX-MS data analysis to overcome the obstacle of limited spatial resolution, providing a unique “quick and cheap” experimental validation to assess models from *ab initio* structure determination methods such as AlphaFold.^42^

## Supporting Information Available

Additional methodological details, including figures and examples (PDF)

## Notes

The scripts and data needed to reproduce the results shown in this article are available on GitHub at the following link: https://github.com/pacilab/exPfact.
