# Supplementary material for: High-Resolution Hydrogen–Deuterium Protection Factors from Sparse Mass Spectrometry Data Validated by Nuclear Magnetic Resonance Measurements
Source: J Am Soc Mass Spectrom. 2022 Apr 6;33(5):813–22. doi: 10.1021/jasms.2c00005 (PMC9074100; doi:10.1021/jasms.2c00005)

Supporting Information

High resolution hydrogen-deuterium protection factors from sparse MS data validated by NMR measurements

Michele Stofella[1,2], Simon P. Skinner[1], Frank Sobott[1], Jeanine Houwing-Duistermaat[3] and Emanuele Paci[1,2*]

1 School of Molecular and Cellular Biology, University of Leeds, LS2 9JT Leeds, United Kingdom

2 Dipartimento di Fisica e Astronomia, Università di Bologna, 40127 Bologna, Italy

3 Dipartimento di Scienze Statistiche, Università di Bologna, 40127 Bologna, Italy

*e.paci@unibo.it

Supplementary Figure 1. When measurements are not affected by experimental error, estimating protection factors is a combinatorial problem. **A.** For an isolated peptide of N residues, the uptake curve is a sum of N exponentials and there are N! possible patterns of protection factors. For N=3, a best fit always extract three rates R1, R2 and R3, however these rates cannot be assigned to specific residues, therefore all the N!=6 assignments (i) to (vi) are possible. **B.** For two overlapping peptides formed by N1=N2=3 residues with Nc=1 common residue as shown, it is possible to unambiguously assign the rate of residue 3, say R3. However, it is not possible to assign rates R1 and R2 to residues 1 and 2 and, analogously, rates R4 and R5 to residues 4 and 5. There are (N1-Nc)!(N2-Nc)!Nc!=4 possible patterns of protection factors, namely options (i) to (iv). **C.** Assignment of rates does not yield to the same result in terms of protection factors. For an isolated peptide of N=2 residues, a best fit always provides rates R1 and R2, e.g. $\mathrm{R1} = 1\mathrm{s}^{-1}$ and $\mathrm{R1} = 10\mathrm{s}^{-1}$. In terms of rates, the N!=2 alternative solutions are $\{\mathrm{R1,R2}\} = \{1, 10\}$ and $\{\mathrm{R2,R1}\} = \{10, 1\}$. If the amino acids corresponding to residues 1 and 2 are different, they have different intrinsic exchange rates, e.g. $k_{int}^{1} = 10\mathrm{s}^{-1}$ and $k_{int}^{2} = 100\mathrm{s}^{-1}$. In terms of protection factors, the 2 alternative solutions are $\left\{\frac{10}{1}, \frac{100}{10}\right\} = \{10, 10\}$ and $\left\{\frac{10}{10}, \frac{100}{1}\right\} = \{1, 100\}$.

A

	1	2	3
i	R1	R2	R3
ii	R1	R3	R2
iii	R2	R1	R3
iv	R2	R3	R1
v	R3	R1	R2
vi	R3	R2	R1

B

	1	2	3		
			3	4	5
i	R1	R2	R3	R4	R5
ii	R1	R2	R3	R5	R4
iii	R2	R1	R3	R4	R5
iv	R2	R1	R3	R5	R4

C

Rates

	1	2
i	R1	R2
ii	R2	R1

⟷

Protection factors

	1	2
i	$K_{int}^{1}/\mathrm{R1}$	$K_{int}^{2}/\mathrm{R2}$
ii	$K_{int}^{1}/\mathrm{R2}$	$K_{int}^{2}/\mathrm{R1}$

Supplementary Figure 2. Protection factors are adjusted to minimize the cost function (Eq. 6 in main text):

$$C(\lambda,\{P_i\}) = \underbrace{\sum_j \sum_k w_{jk}\left[D_j^{pred}(t_k,\{P_i\}) - D_j^{exp}(t_k)\right]^2}_{SSR} + \underbrace{\lambda \sum_i (\ln (P)_{i-1} - 2\ln (P)_i + \ln (P)_{i+1})^2}_{\text{Penalty term}}$$

The cost function consists in a regular term, i.e. the sum of squared residuals (SSR), which depends on the experimental data, and a penalty term, which depends on the estimated parameters (i.e., the protection factors). The penalty term is built to minimize variations between protection factors of neighbouring residues (the functional form in the penalty term is the Newton approximation of the second derivative of a discrete function: $\frac{d^2x}{dt^2} \approx x_{i-1} - 2x_i + x_{i+1}$). The relative contribution of the penalty term with respect to the regular term is determined by penalty constant λ.

We applied leave-one-out cross-validation (CV) to determine the penalty constant λ. The MS dataset was divided into a training dataset formed by 14 of the 15 time points available (for all peptides) and a test dataset composed by the remaining time point. This splitting procedure was repeated by leaving out one time point at a time; as a result, 15 training (and, analogously, test) datasets were generated (Supplementary Figure 2A). Starting from an initial guess on the protection factors (to ensure the reproducibility of the results, the protection factor of every residue except prolines was initialized to 1), the cost function was minimized for each training dataset. The minimized value of the cost function and the estimated set of protection factors was recorded; the sum of the cost function over all training datasets (CV_{train}) was evaluated. The estimated set of protection factors was used to predict the fractional deuterium uptake of the test dataset (i.e., the remaining time points), and the cost function was calculated for every test dataset. The sum of the cost function over all test datasets (CV_{test}) was evaluated. The CV error $CV_{error}(\lambda) = CV_{train}(\lambda) + CV_{test}(\lambda)$ was calculated for λ ranging from 10^{-15} to 10^{-1}. The value of λ corresponding to the minimum CV error was selected: $\lambda = 1 \times 10^{-8}$ (Supplementary Figure 2B).

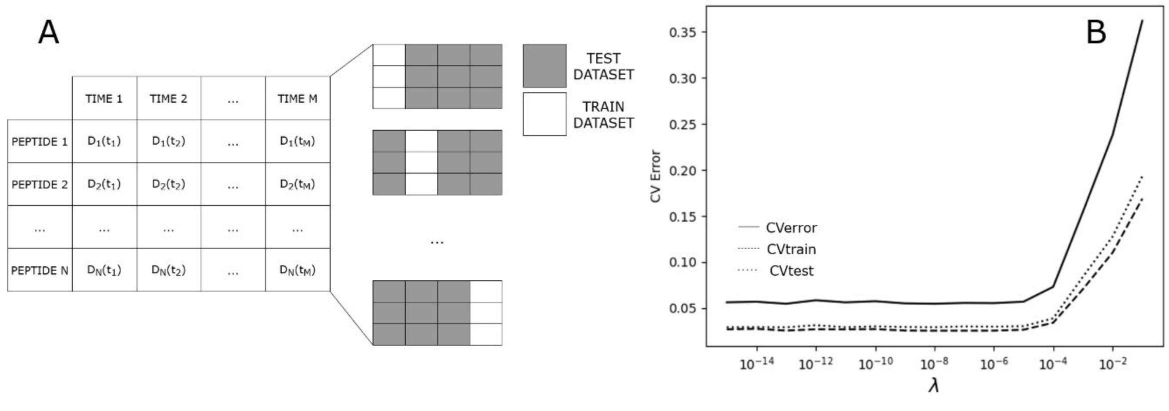

Supplementary Figure 3. Visual representation of the clustering algorithm in 2 dimensions (i.e. for a 2-residue peptide). The histograms of the protection factors that fit deuterium uptake curves (not shown) are shown in panel A. The application of a univariate clustering algorithm would identify two components per amino acid (panel C). The univariate approach provides four approximate solutions for which each amino acid assumes protection factors ~3 and ~7 (namely {3,3},{7,3}, {3,7}, {7,7}). The multivariate algorithm identifies two clusters (panel B), showing that solutions {3,3} and {7,7} are not possible. The clustering algorithm fits a mixture of 1 to 99 2-dimensional Gaussians to the 2-dimensional probability distribution (panel D) and calculates the BIC (Bayesian Information Criterion) associated to a specific number of Gaussian components (insert in panel D). The number of components with highest BIC is 2. Therefore, while the solution is underdetermined, the multivariate approach is less underdetermined than the univariate.

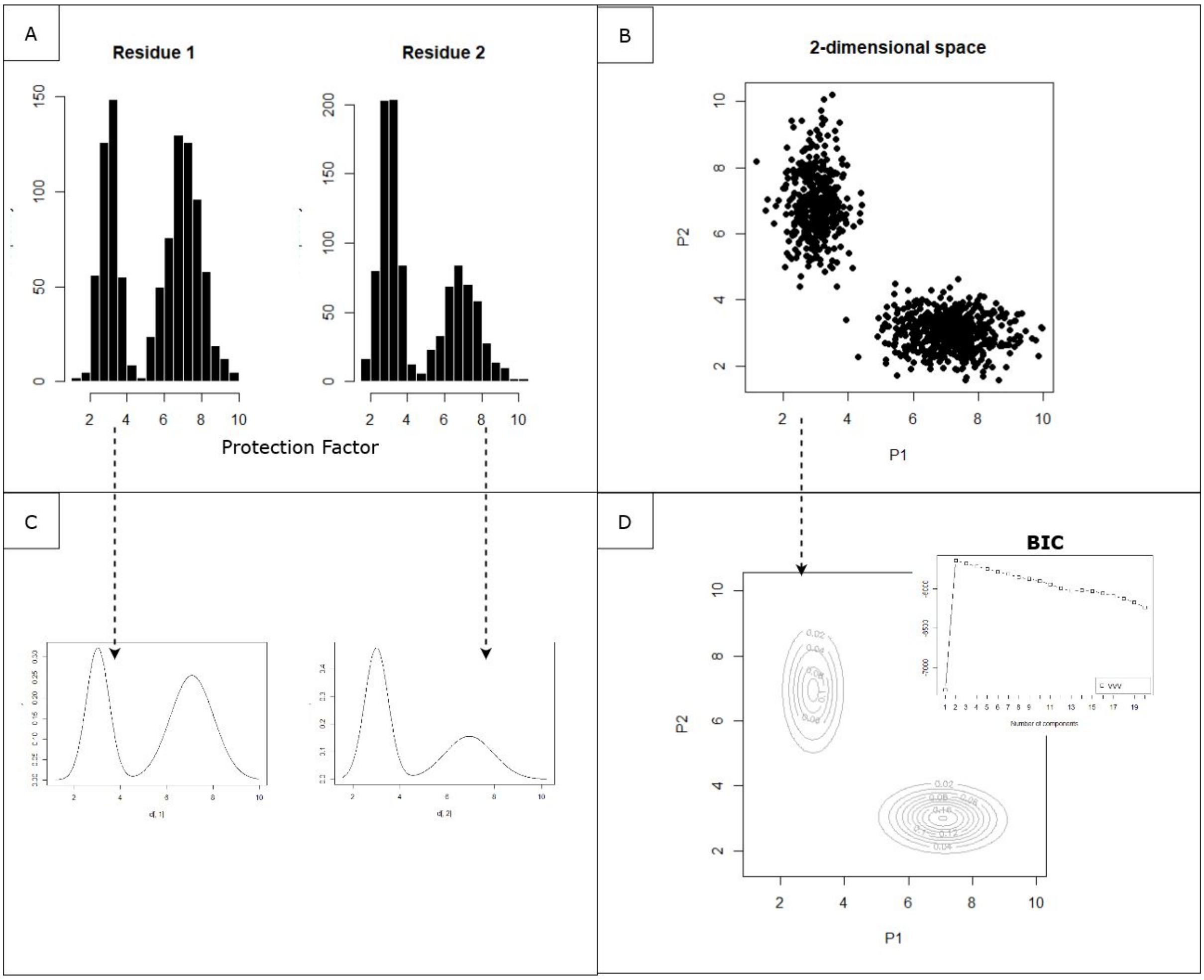

Supplement: Supplementary file 1 — js2c00005_si_001.pdf [file js2c00005_si_001.pdf]
